# 810. Predictors of 30-Day Mortality Among Critically Ill Patients with Candidemia Identified by T2Candida Panel

**DOI:** 10.1093/ofid/ofad500.855

**Published:** 2023-11-27

**Authors:** Kaylee E Caniff, Dana Holger, Kristen Lucas, Matthew A O’Donnell, Ryan K Shields, Angela Loo, Ramy Khem, Nikolai Dahl, Yanina Dubrovskaya, Kassandra Marsh, Ashley L Cubillos, Elisabeth Chandler, Olivia Knack, Susan L Davis, George J Alangaden, Michael J Rybak

**Affiliations:** Anti-Infective Research Lab, Eugene Applebaum College of Pharmacy and Health Sciences, Wayne State University, Royal Oak, MI; Nova Southeastern University, Fort Lauderdale, Florida; Wayne State University, Detroit, Michigan; University of Pittsburgh Medical Center, Pittsburgh, Pennsylvania; University of Pittsburgh, Pittsburgh, PA; New York-Presbyterian Hospital, New York, New York; John Muir Health, Walnut Creek, California; John Muir Health, Walnut Creek, California; NYU Langone Health, New York, New York; NYU Langone Health, New York, New York; Banner North Colorado Medical Center, Loveland, Colorado; Gulf Coast Medical Center, Fort Myers, Florida; AdventHealth Tampa, Tampa, Florida; Wayne State University, Detroit, Michigan; Henry Ford Health, Detroit, Michigan; Eugene Applebaum College of Pharmacy and Health Sciences, Detroit, Michigan

## Abstract

**Background:**

Candidemia is associated with mortality rates exceeding 40%. However, prior studies indicate mortality may be reduced when antifungal therapy is initiated within 12 hours. The T2Candida Panel is a diagnostic assay that detects *Candida* species directly from a whole blood specimen within 3-5 hours (T2 Biosystems, Lexington, MA). The objective of this study is to identify predictors of 30-day mortality in patients with candidemia identified by T2Candida Panel.

**Methods:**

This is a retrospective, multicenter study of critically ill patients with candidemia identified by T2Candida Panel from January 2016 - December 2022. Critically ill patients were defined as those who developed candidemia during an intensive care unit (ICU) stay or within 72 hours of ICU admission or discharge. T2Candida sites were chosen across the United States based on T2Candida utilization. Exclusion criteria were patients < 18 years of age, those with prophylactic indications for antifungal therapy, prisoners and pregnant patients. Multivariate logistic regression was conducted to identify factors associated with 30-day mortality measured from the T2Candida draw time.

**Results:**

There were 171 ICU patients from seven institutions with candidemia identified by T2Candida panel. The mean (standard deviation [SD]) age was 59.7 (14.8) years and 52.1% were male. Mean (SD) APACHE II and Charlson Comorbidity Index scores were 20.6 (7.1) and 4.9 (2.8), respectively. Empiric antifungal therapy was administered to 36.8% of patients and the majority received infectious diseases (ID) consult (92.4%). Echinocandins were the most common agents used for empiric (72.7%) and definitive therapy (62.6%). Overall, 30-day mortality occurred in 36.0% and was not associated with antifungal de-escalation. Administration of empiric therapy (aOR 0.457, 95% CI 0.199-1.054) and ID consult (aOR 0.225, 95% CI 0.056-0.913) were associated with reduced odds of 30-day mortality.

**Conclusion:**

Empiric antifungal administration and ID consult were independently associated with reduced odds of 30-day mortality in patients with candidemia identified by T2Candida Panel. Future studies are needed to evaluate the impact of the T2Candida panel on antifungal stewardship.

**Disclosures:**

**Ryan K. Shields, PharmD, MS**, Allergan: Advisor/Consultant|Cidara: Advisor/Consultant|Entasis: Advisor/Consultant|GSK: Advisor/Consultant|Melinta: Advisor/Consultant|Melinta: Grant/Research Support|Menarini: Advisor/Consultant|Merck: Advisor/Consultant|Merck: Grant/Research Support|Pfizer: Advisor/Consultant|Roche: Grant/Research Support|Shionogi: Advisor/Consultant|Shionogi: Grant/Research Support|Utility: Advisor/Consultant|Venatorx: Advisor/Consultant|Venatorx: Grant/Research Support **Michael J. Rybak, PharmD, PhD, MPH**, Abbvie, Merck, Paratek, Shionogi, Entasis, La Jolla, T2 Biosystems: Advisor/Consultant

